# Improvement of DC vaccine with ALA-PDT induced immunogenic apoptotic cells for skin squamous cell carcinoma

**DOI:** 10.18632/oncotarget.3529

**Published:** 2015-04-20

**Authors:** Jie Ji, Zhixia Fan, Feifan Zhou, Xiaojie Wang, Lei Shi, Haiyan Zhang, Peiru Wang, Degang Yang, Linglin Zhang, Wei R. Chen, Xiuli Wang

**Affiliations:** ^1^ Department of Photomedicine, Shanghai Skin Disease Hospital, Shanghai 200443, China; ^2^ Biophotonics Research Laboratory, Center for Interdisciplinary Biomedical Education and Research, University of Central Oklahoma, Edmond, OK 73034, USA

**Keywords:** dendritic cell, photodynamic therapy, immunogenic apoptosis, 5-aminolevulinic acid, PDT-DC vaccine

## Abstract

Dendritic cell (DC) based vaccines have emerged as a promising immunotherapy for cancers. However, most DC vaccines so far have achieved only limited success in cancer treatment. Photodynamic therapy (PDT), an established cancer treatment strategy, can cause immunogenic apoptosis to induce an effective antitumor immune response. In this study, we developed a DC-based cancer vaccine using immunogenic apoptotic tumor cells induced by 5-aminolevulinic acid (ALA) mediated PDT. The maturation of DCs induced by PDT-treated apoptotic cells was evaluated using electron microscopy, FACS, and ELISA. The anti-tumor immunity of ALA-PDT-DC vaccine was tested with a mouse model. We observed the maturations of DCs potentiated by ALA-PDT treated tumor cells, including morphology maturation (enlargement of dendrites and increase of lysosomes), phenotypic maturation (upregulation of surface expression of MHC-II, DC80, and CD86), and functional maturation (enhanced capability to secrete IFN-γ and IL-12, and to induce T cell proliferation). Most interestingly, PDT-induced apoptotic tumor cells are more capable of potentiating maturation of DCs than PDT-treated or freeze/thaw treated necrotic tumor cells. ALA-PDT-DC vaccine mediated by apoptotic cells provided protection against tumors in mice, far stronger than that of DC vaccine obtained from freeze/thaw treated tumor cells. Our results indicate that immunogenic apoptotic tumor cells can be more effective in enhancing a DC-based cancer vaccine, which could improve the clinical application of PDT-DC vaccines.

## INTRODUCTION

Most current cancer treatment modalities aim at killing tumor cells, directly or indirectly. Since failure of the host immune system's cancer immunosurveillance function is implied in the rise of many if not most cancers [1–3], partial or complete restoration of the immunosurveillance function may prove to be one of the most promising approaches for cancer immunotherapy. In the immune system, dendritic cells (DCs) are the most important antigen-presenting cells (APCs) [4–7]. They are crucial in antigen updating, processing, and presentation to T cells, to induce tumor-specific immune responses [8–9]. Recent studies show that DC-based vaccines obtained through stimulation of DCs by *ex vivo* prepared tumor antigens have yielded promising results in the treatment of cervical cancer, melanoma, and ovarian cancer [10–11].

Photodynamic therapy (PDT) is an established therapy for the treatment of cancerous and other lesions, using a combination of light and photosensitizers to induce damage to the targeted tissues [12]. 5-aminolevulinic acid (ALA), as a hydrophilic, low molecular weight molecule within the heme biosynthesis pathway, is considered as a prodrug. Once ALA is applied to the skin, it accumulates in rapidly proliferating cells and it is converted to its active form, protoporphyrin IX (PpIX), which is a photosensitizer in the PDT reaction [13]. PDT has been shown to induce certain immunological reactions [14–18]. It has been shown that PDT-killed tumor cells tend to induce stronger anti-tumor immunity *in vivo* than tumor cell lysates produced via treatments such as ionizing irradiation or freeze-thaw [19]. Based on these premises, PDT-based tumor vaccines have been developed and have shown good promise in pre-clinical models (and led to Phase I clinical trials along similar lines) [20–21]. In addition, DCs exposed *in vitro* to PDT-treated tumor cell lysates (PDT-DC vaccines) have been used for immunotherapy against mammary cancer and adenocarcinoma in mouse models [22]. In their studies, PDT-DC vaccines or PBS only were injected subcutaneously into the right flank on days 7 and 14 after tumor implantation. Mice treated with PDT-DCs had few, if any, tumors, whereas mice treated with PBS developed tumors. Moreover, PDT-DC vaccination induced an efficient tumor-specific CTL response and resulted in potent stimulation of IFN-γ-secreting CD8^+^ T cells [22].

In a ‘classical’ sense, the most immunogenic cell death pathway is necrosis, since rapid loss of plasma membrane integrity occurring during necrosis is associated with the release of various pro-inflammatory factors [23–26]. On the other hand, apoptosis is often considered to be an immunosuppressive or even tolerogenic cell death process [23–26]. However, our previous study has shown that PDT can cause tumor cells to undergo an immunogenic form of apoptosis and these dying tumor cells can induce an effective antitumor immune response, which is much stronger than the response induced by necrosis [27]. It showed that PDT caused exposure of HSP70 (ecto-HSP70) on the surface of treated cells serving as ‘immunogenic signals’ in opsonisation of cancer cells [28–29]. Damage-associated molecular patterns (DAMPs), HSP70, calreticulin (ecto-CRT), ATP and other molecular targets have recently been identified as crucial elements for immunogenic apoptosis [28–29].

Skin squamous cell carcinoma (SCC), as a tumor of the elderly, has seen its incidence rising due to the increasing life expectancy. SCC manifests as a spectrum of progressively advanced malignancies, ranging from actinic keratosis (AK) to Bowen's disease, invasive SCC and metastatic SCC [30]. Patients with invasive SCCs metastasized to regional nodes constitute a group at high risk for tumor recurrence and cancer-related death [31]. Immunosuppression has been shown to be one of the key prognostic factors for metastasis.

To improve the treatment of SCC, we developed the ALA-PDT-DC cancer vaccine. We specifically focused on the PDT induced apoptotic tumor cells and their effects on potentiating maturation of DCs. We tested the DC vaccine against SCC PECA tumors in mice. Here we present our findings on a strong antitumor immunity induced by the PDT-DC vaccine which was stimulated by immunogenic apoptotic cancer cells. Our study may lead to an improved treatment modality against metastatic cancers.

## RESULTS

### PpIX accumulation in PECA cells

**Figure 1:**
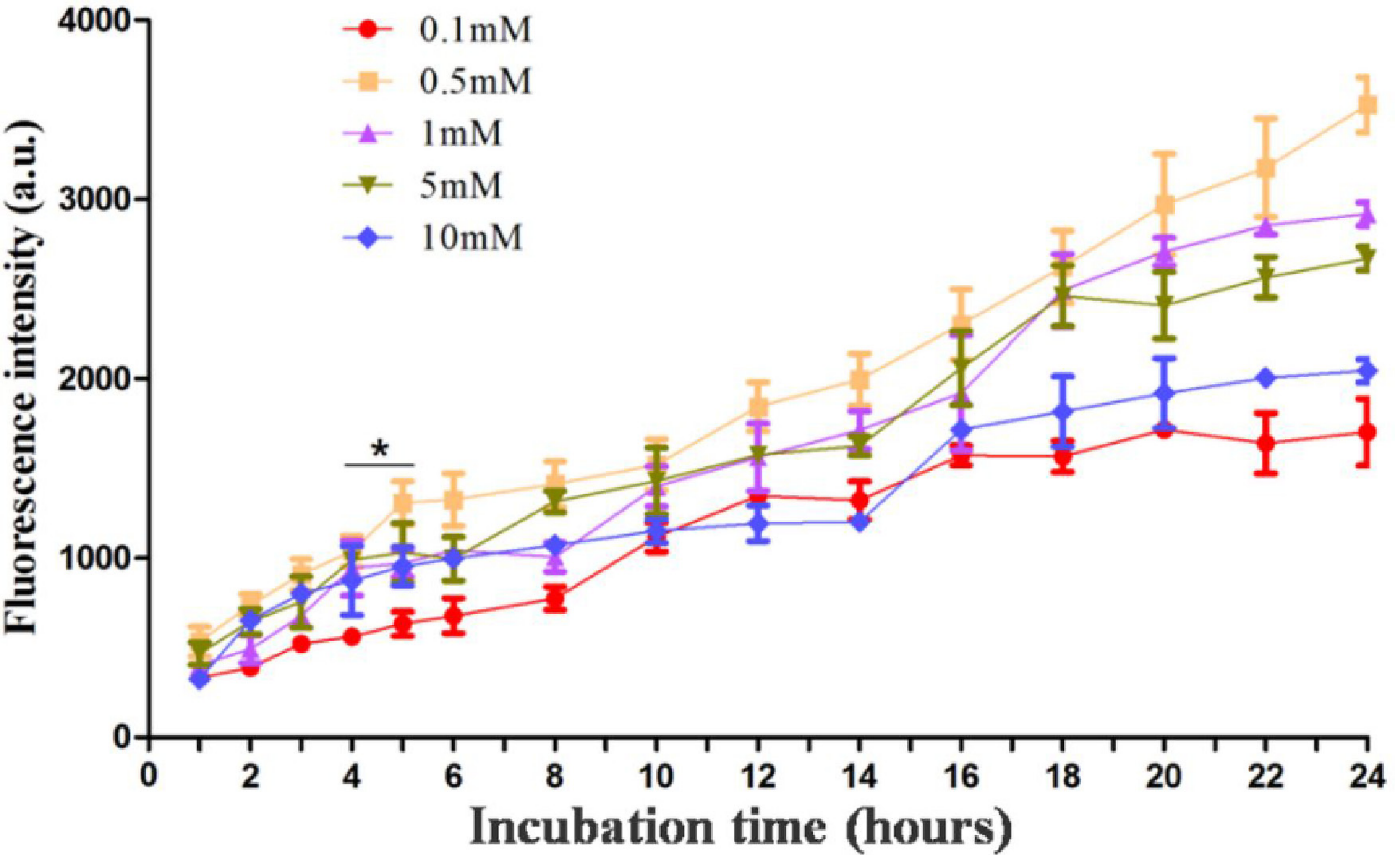
Protoporphyrin IX accumulation in PECA cells PECA cells were incubated with ALA of different concentrations (0.1 to 10 mM) in serum-free medium in the dark with different incubation times (1 to 24 h). PpIX fluorescence emission from the cells was detected using a microplate reader (*n* = 5). PpIX accumulation in PECA cells increased with the incubation time. PpIX fluorescence in cells incubated with 0.5 mM ALA was higher than that from cells incubated with other ALA concentrations. PpIX accumulation in PECA cells incubated with 0.5 mM ALA for 5 h was higher than that for 4 h (*p* < 0.05), but close to that for 6 h (*p* > 0.05).

To investigate ALA-mediated PpIX accumulation, PECA cells were incubated with ALA of different concentrations (0.1 to 10 mM) in a serum-free medium in the dark with different incubation times (1–24 h). At designated time points, PpIX fluorescence emission from the PECA cells was detected using a microplate reader. The fluorescence intensity increased with the incubation time (Figure 1). PpIX accumulation in cells incubated with 0.5 mM ALA was higher than that incubated with other ALA concentrations. Furthermore, PpIX production in PECA cells incubated with 0.5 mM ALA for 5 h was higher than that for 4 h (*p* < 0.05), but close to that for 6 h (*p* > 0.05). In the current clinical practice, the best incubation time for ALA is approximately 3–6 h. Therefore, 0.5 mM ALA and 5 h incubation time were selected as the optimal parameters for the PDT treatment.

### Cell death under different treatments

**Figure 2:**
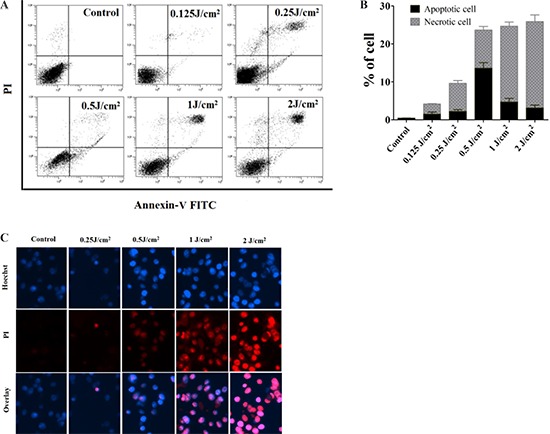
Death of PECA cells after ALA-PDT **A.** and **B.** FACS analysis for cell death. PECA cells were stained with Annexin-V-FITC/PI after ALA-PDT with different light doses (0.125 to 2 J/cm^2^), 1 h after treatment (*n* = 5). Cell death increased with light dose. Under 0.5 J/cm^2^ light dose, the proportion of apoptotic PECA cells reached maximum, while 2 J/cm^2^ light dose mainly induced necrotic cells. **C.** Cell death revealed by (Hoechst) /PI staining 1 h after different treatments, with untreated cells as a control. ALA-PDT at 0.5 J/cm^2^ mainly induced apoptosis while ALA-PDT at 2 J/cm^2^ mainly induced necrosis.

To confirm cell death induced by PDT with different light doses, FACS and Hoechst/PI staining were used. PECA cells were incubated with 0.5 mM ALA for 5 h in the dark and were irradiated with different light doses (0.125 to 2 J/cm^2^). Apoptosis and necrosis of PDT treated PECA cells were determined by flow cytometry 1 h after treatment. FACS showed that PDT induced cell death increased with light dose (Figure 2A and 2B). Under 0.5 J/cm^2^ fluence, the proportion of apoptotic PECA cells reached maximum, while 2 J/cm^2^ fluence mainly induced necrosis (Figure 2A and 2B). The Hoechst and PI staining of PDT treated tumor cells (Figure 2C) confirmed these results.

### Morphology of DCs after interaction with PDT-PECA cells

**Figure 3:**
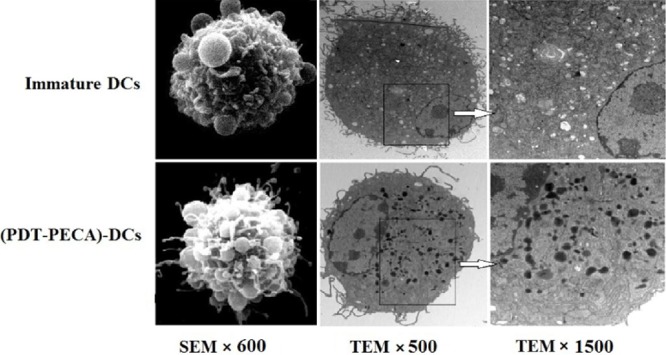
Morphology of DCs detected by scanning electron microscopy (SEM) and transmission electron microscopy (TEM) Top Panel: SEM and TEM images of immature DCs. Bottom Panel: SEM and TEM images of DCs after stimilation by PDT-treated PECA cells. PECA cells were treated by PDT (0.5 mM ALA, 0.5 J/cm^2^). Six hours after PDT treatment, PECA cells were incubated with imDCs for 24 h. Then DCs were visualised by SEM (left column, ×600), TEM (×500) (middle column) and TEM (×1500) (right column). The enlargement of dendrites and increase of lysosomes were clearly seen in DCs after stimulation by PDT-treated PECA cells.

DC growth was monitored daily using contrast microscopy. On day 1, the cells were most spherical, resembling that of isolated bone marrow mononuclear cells. By day 3, the number of bone marrow mononuclear cells increased, and the presence of immature bone marrow cell colonies was observed. On day 7, most cells were suspended and demonstrated dendritic cell morphology, with characteristic irregular nuclei and short cytoplasmic protrusions (data not shown). On day 7, the immature DCs were incubated with PECA cells treated with ALA-PDT at a light dose of 0.5 J/cm^2^, the apoptotic dose. After 24 h incubation, the DCs were visualized by scanning electron microscopy (SEM) and transmission electron microscopy (TEM). The DCs demonstrated polygonal, oval, or round shapes. DCs incubated with PDT-treated tumor cells showed a larger number of uneven protrusions and dense lysosomes, compared with immature DCs, as shown in Figure 3. These results indicated that the morphology maturation (enlargement of dendrites and increase of lysosomes) of DCs could be potentiated by ALA-PDT treated tumor cells.

### Phenotypic maturation of DCs after interaction with PDT-PECA cells

**Figure 4:**
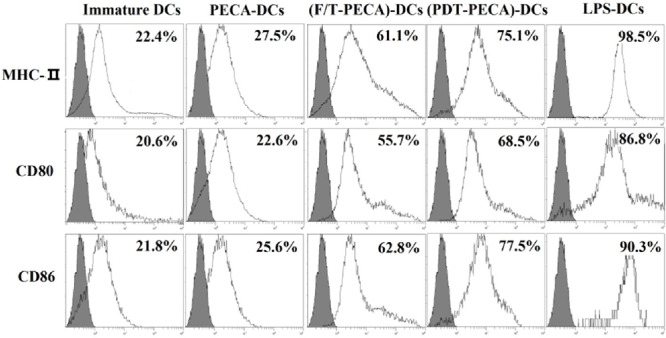
Phenotypic maturation of DCs PECA cells were treated by PDT (0.5 mM ALA, 0.5 J/cm^2^) or three cycles of freeze thaw (F/T). Six hours after treatment, the treated and untreated PECA cells were collected and incubated with imDCs for 24 h, followed by the detection of MHC-II, CD80 and CD86 on the surface of DCs. Unstimulated, immature DCs were used for a negative control and DCs incubated with LPS were used for the positive control. PDT-treated PECA cells have a much greater ability to upregulate expression of CD80, CD86, and MHC-II molecules on the surface of DCs than untreated PECA cells or F/T treated PECA cells.

PECA cells were treated by ALA-PDT (0.5 mM ALA, 0.5 J/cm^2^) or three cycles of freeze thaw (F/T). Six hours after treatment, the cells were collected and incubated with imDCs for 24 h. As a positive control, imDCs were stimulated by LPS, with untreated imDCs as a negative control. Surface expression of CD80, CD86, and MHC-II molecules of DCs was characterized by FACS. The PDT treated PECA cells caused the increase of the expression of CD80, CD86, and MHC-II molecules on the surface of DCs, markedly higher than that caused by untreated PECA cells and F/T PECA cells, as shown in Figure 4. The expression of the molecules on DCs induced by PECA cells treated by PDT was significantly higher than that by untreated PECA cells or PECA cells treated by freeze thaw (*p* < 0.05).

### Functional maturation of DCs stimulated by PDT-treated PECA cells

**Figure 5:**
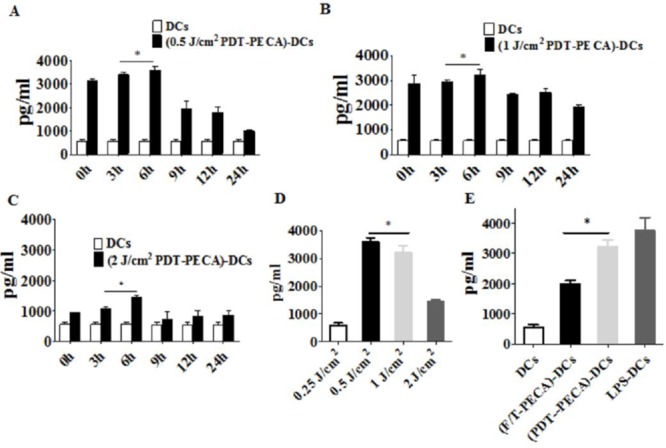
Secretion of IFN-γ from DCs Secretion of IFN-γ from DCs after incubation with PECA cells treated by ALA-PDT with 630 nm LED light at doses of 0.5 J/cm^2^ **A.** 1 J/cm^2^ **B.** or 2 J/cm^2^ **C.** at different times (0 to 24 h) after light irradiation, with untreated imDCs as a negative control. **D.** PECA cells were treated by ALA-PDT with different light doses (0.25 to 2 J/cm^2^). Six hours after treatment, PECA cells were collected and incubated with imDCs for 24 h and IFN-γ secreted from the DCs were quantified. **E.** PECA cells treated by PDT (0.5 mM ALA, 0.5 J/cm^2^) or three cycles of freeze thaw (F/T). Six hours after treatment, PECA cells were incubated with imDCs for 24 h, followed by measurement of IFN-γ secreted form DCs. Secretion of IFN-γ form untreated imDCs was used as a negative control and secretion of IFN-γ form DCs after incubation for 24 h with LPS was used as a positive control.

**Figure 6:**
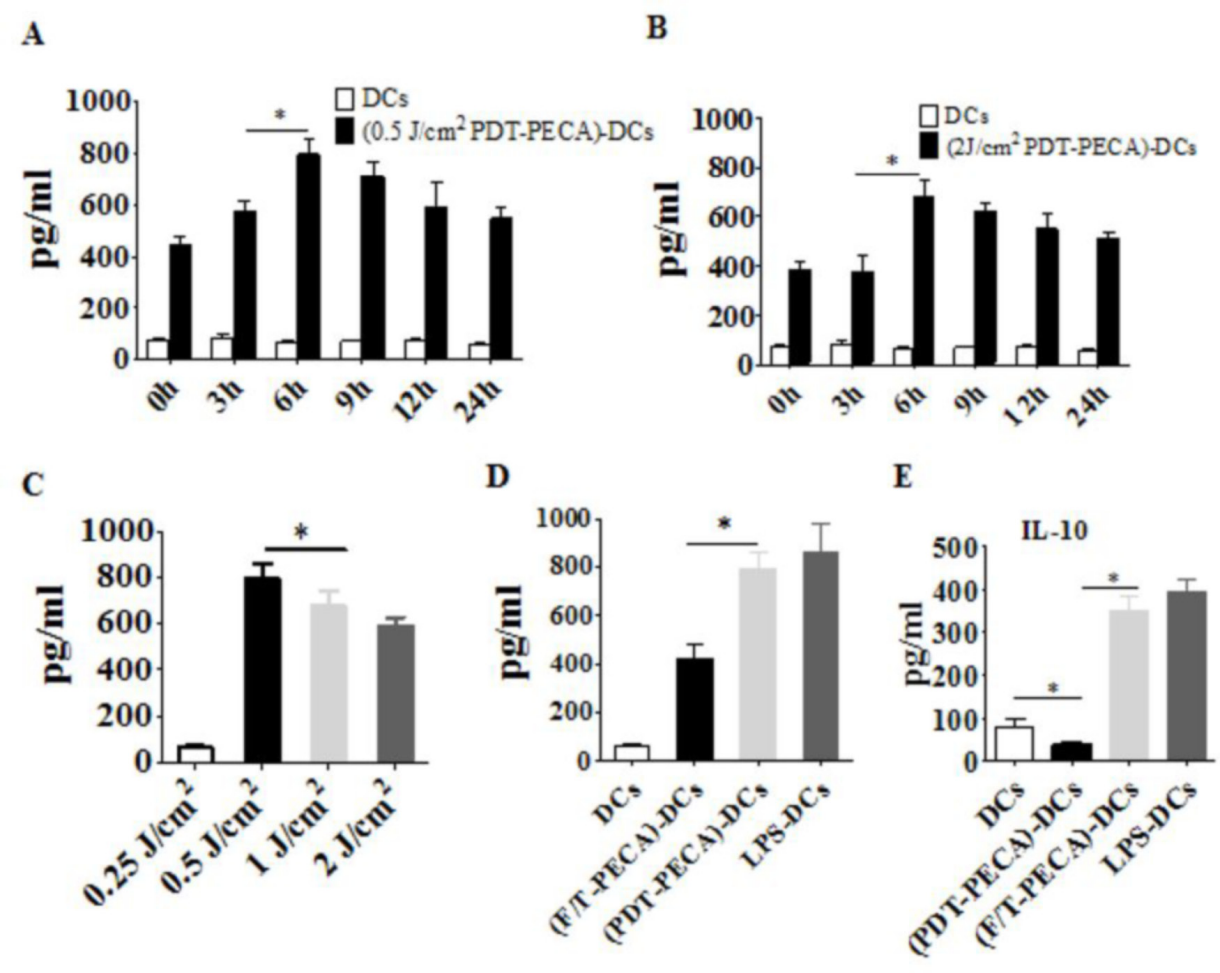
Secretion of IL-12 and IL-10 from DCs Secretion of IL-12 from DCs after incubation with PECA cells treated by ALA-PDT with 630 nm LED light at doses of 0.5 J/cm^2^ **A.** or 2 J/cm^2^ **B.** at different times (0 to 24 h) after light irradiation, with unstimulated imDCs as a control. **C.** PECA cells were treated by ALA-PDT with different light doses (0.25 to 2 J/cm^2^). Six hours after treatment, PECA cells were collected and incubated with imDCs for 24 h and IL-12 secreted from the DCs were quantified. **D.** and **E.** PECA cells treated by PDT (0.5 mM ALA, 0.5 J/cm^2^) or three cycles of freeze thaw (F/T). Six hours after treatment, PECA cells were collected and incubated with imDCs for 24 h and IL-12 (D) or IL-10 (E) was measured. Secretion of IL-12 or IL-10 form unstimulated imDCs was used as a negative control and secretion of IL-12 or IL-10 form DCs after incubation for 24 h with LPS was used as a positive control.

The functional state of DCs incubated with PECA cells under different treatments was analyzed by assaying supernatants from DC cultures for the presence of IFN-γ, IL-12 and IL-10. Increased levels of IFN-γ and IL-12 secretion from DCs were detected after incubated with PECA cells treated by PDT with different light doses (0.25 to 2 J/cm^2^) or freeze-thaw (Figure 5 and Figure 6). The recovery time of PECA cells after PDT affected functions of DCs, as shown in Figure 5, with most cytokines reaching the highest levels when DCs were incubated with PDT-treated PECA cells after 6 h recovery (Figure 5 and Figure 6). It was also found that apoptotic tumor cells induced by PDT (0.5 J/cm^2^) stimulated a higher level of IFN-γ and IL-12 than that stimulated by necrotic cells induced by PDT (2 J/cm^2^) or by freeze thaw. F/T-PECA cells stimulated the production of IL-10, an immunosuppressive cytokine, whereas ALA-PDT-treated cells reduced the production of IL-10 (Figure 6E).

### T cell proliferation stimulated by DCs

**Figure 7:**
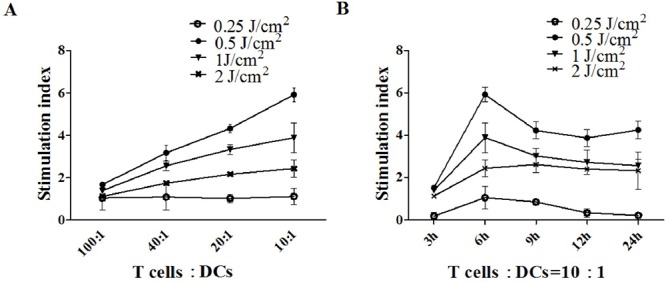
T cell proliferation stimulated by DCs **A.** PECA cells were treated by ALA-PDT with different light doses (0.25 to 2 J/cm2). Six hours after treatment, PECA cells were incubated with imDCs for 24 h. Then T cells were incubated with collected DCs in different ratios (100:1, 40:1, 20:1, 10:1) for 72 h (*n* = 5). **B.** PECA cells were treated by PDT with different light doses (0.25 to 2 J/cm2). At different times (3 to 24 h) after PDT, PECA cells were incubated with imDCs for 24 h. Then T cells were incubated with collected DCs at a ratio of 10:1. Untreated T cell were used for a negative control, T cell proliferation was quantified by CCK-8 assay. ALA-PDT-treated PECA cells enhanced the capability of DCs to induce T cell proliferation. DCs stimulated by PDT induced apoptotic PECA cells are more potent stimulators of T cell proliferation than DCs stimulated by PDT induced necrotic PECA cells.

PECA cells were treated by PDT with different light doses (0.25 to 2 J/cm^2^). At different times (3 to 24 h) after treatment, cells were collected and incubated with DCs for 24 h. Then DCs were incubated with T cells in different Responder to Stimulator ratios (100:1, 40:1, 20:1, 10:1). CCK-8 assay was performed to detect T cell proliferation. DCs caused significant T cell proliferation as a function of the ratio. The ALA-PDT-treated PECA cells enhanced capability of DCs to stimulate T cell proliferation. Furthermore, DCs stimulated by PDT induced apoptotic PECA cells (PDT, 0.5 J/cm^2^, 6 h after treatment) are more potent stimulators of T cell proliferation than DCs stimulated by PDT induced necrotic PECA cells (Figure 7).

### Anti-tumor immunity of the PDT-DC vaccine

**Figure 8:**
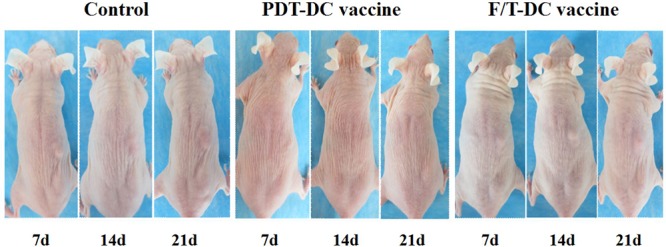
Anti-tumor immunity of PDT-DC vaccine PECA cells were treated with ALA-PDT (0.5 mM ALA, 0.5 J/cm^2^) or three cycles of freeze thaw (F/T). Six hours after treatment, the cells were collected and incubated with imDCs for 24 h. SKH-1 mice were randomly divided into three groups (10 per group), immunized with PDT-DC vaccine, F/T-DC vaccine or PBS. Mice were then challenged with viable PECA cells in the right flank 7 days after the third immunization. No tumors were seen at the challenge site in the PDT-DC vaccine group, while all mice immunized with PBS or the F/T-DC vaccine experienced tumor growth after tumor cells challenge.

To test the anti-tumor immunity of the ALA-PDT-DC vaccine, we immunized SKH-1 mice with DCs after stimulation by PDT treated PECA cells. As negative and positive controls, mice were immunized with PBS or DCs after stimulation by freeze/thawed PECA cells, respectively. The immunization was performed three times; then the mice were challenged with viable PECA cells. Twenty-one days after injection with viable PECA cells, no tumors were observed in the PDT-DC vaccine group, while all mice immunized with PBS or F/T-DC vaccine experienced tumor growth after tumor cells challenge (Figure 8).

## DISCUSSION

Immunotherapy is now widely recognized as a promising cancer treatment modality. Since DCs are important immune cells for uptaking, processing, and presenting tumor antigens, DC-based vaccines are an attractive approach to treat cancers [32–34]. Clinical trials provide evidence that DC vaccines can elicit immunological responses, yet few complete tumor remissions have been reported [22].

Recently, a novel therapy using laser-assisted photothermal interaction and adjuvant-enhanced immunostimulation has been developed [35]. Its pre-clinical and preliminary clinical results showed promising outcomes [36–39]. It utilizes a local intervention to induce a systemic anti-tumor immunity, using as its mechanism *in situ* autologous whole-cell vaccination. It is expected that this thermal-vaccine approach could be significantly enhanced by DC-derived external vaccines. Here, we intend to investigate the enhancement of DC vaccines.

PDT is an effective method for the treatment of tumors. ALA-PDT has the advantages of minimal invasiveness, better aesthetic outcomes, low morbidity, minimal functional disturbance, good tolerance, and the ability to be used repeatedly at the same site [40]. It has been demonstrated that PDT can generate a depot of tumor-associated antigens (TAAs) and, alone or in combination with other modalities, elicit antitumor immune responses. We have used an SCC mouse model to study therapeutic and immune effects of ALA-PDT and found that topical ALA-PDT could induce quick apoptosis, inhibit SCC growth and shrink tumor volume [41]. In our previous studies, immunohistochemistry assays showed the numbers of DCs, CD4+ and CD8+ T cells distributed in the tumor tissues increased gradually after topical PDT. In addition, there was a marked increase in TNF-α expression after PDT [41].

In clinical practice, the outcomes of ALA-PDT for treatment of tumors depend on ALA concentration, ALA incubation time, and light dose. Similarly, these parameters also determine the cytotoxicity of ALA-PDT on tumor cells. When we studied the kinetics of PpIX fluorescence in PECA cells, it was found that PpIX production in PECA cells increased with the incubation time (Figure 1). This result is consistent with other studies [42–43]. We also found that PpIX production in PECA cells didn't depend on ALA concentration. In our study, the PpIX fluorescence emission from cells incubated with 0.5 mM ALA were higher than from cells incubated with other ALA concentrations. It is assumed that, on the cell membrane, there may be some drug transporters, e.g., P-glycoprotein (P-gp), multidrug resistance related protein (MRP), to efflux of chemical compounds [44–45]. After incubation with ALA at 0.5 mM for 5 h, the PpIX production in PECA cells was higher than that incubated for 4 h (*p* < 0.05), but close to that for 6 h (*p* > 0.05), as shown in Figure 1. In the current clinical practice, the best incubation time for ALA is approximately 3–6 h. Therefore, in our *in vitro* studies, 0.5 mM ALA and 5 h incubation were selected. It is reported that the optimal ALA concentration for A431 (human skin squamous cell carcinoma cell line) cells is 1 mM ALA. Studies showed that kinetics of ALA-induced PpIX fluorescence varied depending on the type of cells [44–45].

It is well known that ALA-PDT is capable of inducing tumor cell death via both apoptotic and necrotic pathways. This study showed that PDT induced cell death increased with light dose (Figure 2A and Figure 2B). Under 0.5 J/cm^2^ fluence, the proportion of apoptotic PECA cells reached maximum, while 2 J/cm^2^ fluence mainly induced PECA cells to undergo necrosis (Figure 2). The concept of ‘immunogenic apoptosis’ has emerged in recent years [28–29]. It has been proposed that apoptotic tumor cells are more effective than necrotic tumor cells in inducing antitumor immune responses [27, 46].

Immature DCs are potent phagocytes but poor T cell stimulators. Therefore, DC maturation is a critical step in the induction of the immune response. We observed that morphology maturation of DCs could be potentiated by ALA-PDT treated apoptotic PECA cells, including enlargement of dendrites and increase of lysosomes (Figure 3). These changes can make DCs capable of capturing and processing tumor antigens.

The ability of DCs to induce the immune response depends on the morphology maturation, expression of specific markers, such as CD80, CD86, and MHC-II. In this study, it was demonstrated that ALA-PDT-treated PECA cells induced phenotypic maturation of DCs, as indicated by up-regulation of MHC class II and co-stimulatory CD80 and CD86 molecules (Figure 4). The surface expression of these molecules was higher than that induced by freeze-thawed PECA cells.

To get further insight into the functional status of DCs, we observed cytokines secreted from DCs after stimulation by ALA-PDT-treated PECA cells. We found that DCs exposed to ALA-PDT-treated cancer cells displayed a distinguished pattern of functional activation characterized by IFN-γ^high^, IL-12^high^, and IL-10^absent^ (Figure 5 and Figure 6). Interestingly, LPS and especially freeze-thawed cells stimulated the production of IL-10 (Figure 6), whereas ALA-PDT-treated cells reduced stimulate the production of this immunosuppressive cytokine.

T cell immunity is initiated by interaction of naive T cells with DCs. T cell proliferation is a critical step in the induction of the immune response. Mature DCs are potent T cell stimulators. In this study, the abilities of DCs to induce T cell proliferation were also investigated. We found that PDT-induced apoptotic tumor cells are more capable of potentiating functional maturation of DCs (enhanced capability to secret IFN-γ and IL-12 and to induce T cell proliferation) than PDT-treated or freeze/thaw-treated necrotic tumor cells, particularly using a light dose of 0.5 J/cm^2^ with an ALA concentration of 0.5 mM (Figure 7). DCs co-cultured with PECA cells, with a 6 h recovery after PDT, produced a significantly higher level of IFN-γ and IL-12 and had the most potential to induce T cell proliferation (Figure 7). The mechanism is not clear and more research is required to ascertain the link between the immunogenicity of PDT-treated tumor cells and the recovery time after PDT.

In this study, we developed DC-based SCC vaccine using PDT induced apoptotic PECA cells (using a light dose of 0.5 J/cm^2^ with an ALA concentration of 0.5 mM, incubated for 5 h, with a 6 h recovery after PDT treatment). The anti-tumor immunity of the ALA-PDT-DC vaccine was tested in SKH-1 mice. Protection against tumor growth at the challenge site was interpreted as a sign of successful priming of the adaptive immune system. ALA-PDT-DC vaccine prevented SCC growth seen in the non-immunized mice [Figure 8]. By contrast, freeze-thawed PECA cells are able to activate DCs to express IFN-γ and IL-12, which are critical to the development of a cellular immune response; however, most of the mice immunized with DC vaccine obtained from F/T treated cells experienced tumor growth after challenge. It may be due to the fact that F/T DCs simulated abundant production of IL-10, which is considered to be an immunosuppressive cytokine.

In conclusion, this study shows that ALA-PDT treated tumor cells can stimulate the maturations of DCs, including morphology maturation, phenotypic maturation, and functional maturation. Most interestingly, PDT induced apoptotic tumor cells are more capable of potentiating maturation of DCs than PDT-treated or freeze/thaw-treated necrotic tumor cells. ALA-PDT-DC vaccine based on apoptotic tumor cells can activate the adaptive immune system more effectively, providing protection against skin squamous cell carcinoma in mice, far stronger than that of F/T-DC vaccine. Our results indicate that immunogenic apoptotic cells induced by ALA-PDT can enhance DC vaccine for SCC.

## MATERIALS AND METHODS

### Animal and cell line

SKH-1 mice (female, 8 weeks old, hair-less, immunocompetent), weighing approximately 30 g, were provided by Shanghai Public Health Clinical (Shanghai Certificate number 2010–0024, China). PECA cell line was obtained from the Cell Lines Service (Germany). PECA cells were maintained in RPMI 1640 medium supplemented with 10% fetal bovine serum (FBS), penicillin (100 IU mL^−1^) and streptomycin (100 μg mL^−1^).

### Materials and reagents

RPMI 1640 cell culture medium, PBS, and penicillin/streptomycin were obtained from Hyclone (Thermo Scientific, Waltham, Massachusetts, USA). Fetal bovine serum (FBS) was obtained from GIBCO (California, USA). ALA hydrochloride powder was obtained from Shanghai Fudan-Zhangjiang Bio-Pharmaceutical Co, Ltd (Shanghai, China). The following chemicals and commercially available assay kits were used: apoptosis detection kit Annexin-V-FITC/PI (R&D Systems, Minneapolis, MN), rabbit anti-Mouse CD80-FITC, rabbit anti-Mouse CD86-FITC, rabbit anti-Mouse MHC-II-PE, rat IgG2a K Isotype Control FITC, Armenian Hamster IgG Isotype Control PE (eBioscience, USA), Hoechst 33342/PI kit (Beyotime Institute of Biotechnology, China), Mouse IFN-γ, IL-12 and IL-10 ELISA Kit (R&D Systems), and MTT assay Kit (Sigma-Aldrich, St Louis, MO, USA).

### PpIX fluorescence measurement

Fresh PECA cells (1 × 10^5^ cells/well) were seeded in 96-well black wall plates (Corning Inc, Corning, NY, USA) and cultured in RPMI 1640 medium supplemented with 10% FBS, penicillin (100 IU mL^− 1^), streptomycin (100 μg mL^− 1^) for 24 h. Attached cells were washed twice with PBS and then incubated with ALA of different concentrations (0.1 to 10 mM) in 100-μL serum-free medium in the dark with different incubation times (1 to 24 h). PpIX fluorescence emission from the cells was detected using a microplate reader (Synergy 2 multi-mode microplate reader; BioTek, Winooski, VT, USA). The fluorescence excitation wavelength was set at 400 nm and the emission wavelength at 635 nm. Data were corrected for autofluorescence background (*n* = 5).

### PDT treatment of tumor cells

For photodynamic therapy, PECA cells growing in 35 mm Petri dishes were incubated in the dark with 0.5-mM ALA in serum-free medium for 5 h, rinsed twice with PBS, and irradiated by a LED light (630 nm, Philips, Netherlands) at a power density of 10mW/cm^2^, with different fluences (0.125 to 2 J/cm^2^). PECA cells without treatment were used as a negative control. For fluorescence microscopy analysis, cells were stained with Hoechst 33342 for 10 min at room temperature and then stained with PI at 4^°^C, and washed twice with PBS. The cell samples were visualized under a Nikon fluorescent microscope (mercury lamp, Ex. 330–380 nm, Em. BA 435 nm). For FACS analysis, cells were cultured with 50 ng/ml PI and 2 μg/ml Annexin V-FITC, 1 h after PDT, with cells of no treatment as control. Fluorescent emission of FITC was measured at 515–545 nm and that of DNA-PI complexes at 565–606 nm, using a FACScan flow cytometer (Becton Dickinson, Mountain View, CA) with an excitation at 488 nm.

### Preparation of dendritic cells

DCs were isolated and cultured according to the method of Inaba et al [47]. Briefly, DCs were obtained from bone marrow precursors by flushing femur, tibia, and humerus bones of 8-week old SKH-1 mice with RPMI-1640. Red blood cells were lysed with Tris-NH4Cl. Cells (1 × 10^7^ cells/well) were then cultured in 6-well plates in fresh complete medium, containing RPMI-1640 supplemented with 10% FBS, 20 ng/ml granulocyte macrophage colony-stimulating factor (GM-CSF, PeproTech) and 10 ng/ml interleukin-4 (IL-4, PeproTech). After 48 h, the culture medium was removed and fresh medium was added. On day 5, 50% of medium was replaced with a fresh medium. Loosely adherent cells (immature dendritic cells, imDCs) collected on day 7 were used for the experiments.

### Morphological assessment of DCs

PECA cells were incubated in the dark with 0.5 mM ALA in serum-free medium for 5 h, rinsed twice with PBS, and irradiated by a LED light (630 nm, 10 mW/cm^2^) at a fluence of 0.5 J/cm^2^. Six hours after treatment, the PECA cells were incubated with imDCs at a ratio of 1:20 (imDCs:PECA) for 24 h, with imDCs without treatment as control. The DCs were collected, rinsed three times with cold PBS, and fixed in 2% glutaraldehyde for 2 h. The morphology of DCs was examined under scanning electron microscopy and transmission electron microscopy (CM-120; Philips).

### Flow cytometric analysis of DCs

PECA cells without treatment or treated by ALA-PDT (0.5 mM ALA, 0.5 J/cm^2^) or three cycles of freeze thaw (F/T) were incubated with imDCs at a ratio of 1:20 (imDCs:PECA) for 24 h. Unstimulated, immature DCs were used for a negative control and DCs incubated with 4 ug/ml LPS for 24 h were used for a positive control. After detachment and washing, the DCs were stained with the following antibodies: anti-Mouse CD80-FITC, anti-Mouse CD86-FITC, anti-Mouse MHC-II-PE according to manufacturer's instructions. After the antibody staining, the cells were washed and analyzed with a FACScan flow cytometer (Becton Dickinson, Mountain View, CA).

### Detection of cytokine from DCs

PECA cells were treated by ALA-PDT with different light doses (0.25 to 2 J/cm^2^) or three cycles of freeze thaw (F/T). PECA cells were collected at different times after treatment (0 to 24 h). To detect cytokines (IFN-γ, IL-12 and IL-10) secreted from DCs, imDCs were incubated with the treated and untreated PECA cells at a ratio of 1:20 (imDCs:PECA) in 24-well tissue culture plates. After 24 h of incubation, the supernatants were collected and divided into different groups for ELISA detection according to manufacturer's instructions. Immature DCs without treatment were used for a negative control and DCs incubated with 4 ug/ml LPS for 24 h were used for a positive control.

### Mixed leukocyte reaction (MLR)

PECA cells treated by ALA-PDT with different light doses (0.25 to 2 J/cm^2^). Six hours after PDT, cells were collected and incubated with imDCs at a ratio of 1:20 (imDCs:PECA) for 72 h. For mixed lymphocyte reaction (MLR), the DCs (2 × 10^6^/ml) after stimulation by PDT-PECA cells were treated with mitomycin C (25 g/ml, Sigma) for 30 min to prevent further mitogenic activity of DCs. Purified T cells from splenocytes of SKH-1 mice were used as responder cells, growing in 96-well culture plates (2 × 10^6^/ml, 100 ul/well). Responder cells were cultured with stimulator cells in different Responder to Stimulator ratios (100:1, 40:1, 20:1, 10:1) for 72 h (*n* = 5). Untreated T cells were used for a negative control. Besides, at different times after treatment (3 to 24 h), the PECA cells were collected and incubated with imDCs for 72 h. Then the DCs were used as stimulator cells. Responder cells were cultured with stimulator cells at a ratio of 1:10 (DCs:T) for 72 h (*n* = 5). T cell proliferation was quantified by CCK-8 assay according to manufacturer's instructions. The final calculation of cell stimulation index (SI) = (OD value of experimental wells-OD value of control wells)/OD value of control wells.

### Detection of PDT-DC vaccine induced *in vivo* immune response

PECA cells were treated by ALA-PDT (0.5 mM ALA, 0.5 J/cm^2^) or three cycles of freeze thaw (F/T). Six hours after treatment, the cells were collected and incubated with imDCs for 24 h. To detect DC vaccine induced immune response *in vivo*, female SKH-1 mice, age 6–8 weeks were randomly divided into three groups (10 per group). The mice were immunized with PDT-DC vaccine or F/T-DC vaccine. Approximately 5 × 10^6^ DCs in 0.5 ml PBS were injected subcutaneously into the left flank of mice. Immunization was done three times with a 10-day interval. Control mice were injected with 0.5 ml PBS. Mice were then challenged with 6 × 10^5^ viable PECA cells in the right flank 7 days after the third immunization. Following the challenge, the mice were monitored every day.
